# The importance of water quality in classifying basic water services: The case of Ethiopia, SDG6.1, and safe drinking water

**DOI:** 10.1371/journal.pone.0248944

**Published:** 2021-08-05

**Authors:** Shibabaw Tadesse Gemeda, Emily Springer, Sirak Robele Gari, Solomon Melake Birhan, Hailu Tolasa Bedane

**Affiliations:** 1 Ethiopian Institute of Water Resources, Addis Ababa University, Addis Ababa, Ethiopia; 2 Social Justice & Human Rights, School of Social and Behavioral Sciences, New College of Interdisciplinary Arts and Sciences, Arizona State University, Glendale, Arizona, United States of America; 3 Environmental Health Department, Wollo University, Dessie, Ethiopia; 4 Doctor of Transformational Leadership Programme, Bakke Graduate University, Dallas, Texas, United States of America; Indian Institute of Technology Bombay, INDIA

## Abstract

**Introduction:**

Sustainable Development Goal (SDG) 6 aims to coordinate international efforts toward “clean water and sanitation.” However, water contaminated with pathogenic bacteria or thermotolerant coliforms (TTC) will not achieve the SDG target of clean water in the lives of people around the world. The aim of this study is to assess the water quality parameters of basic water services in Amhara and Afar regions of Ethiopia as well as the role and importance of local managerial committees in ensuring basic water functionality.

**Methods:**

This mixed methods research, conducted in January-June 2019, sampled 22 districts from food-insecure areas in the Amhara and Afar regions of Ethiopia. From the 22 districts, which represent nearly one third of all districts in each region, 111 water services classified as “basic” were randomly selected. For each selected water service, research included: water quality sample testing, visual observation of water services, interviews and focus group discussions with the associated water managerial committee members. Descriptive statistics frequency, percent, mean, median, standard deviations, normal tables, cross-tables and graphs are used to present the data.

**Results:**

Although the international water standard for thermotolerant coliform (TTC) levels is 0 CFU/100ml, in our sample of 111 water services, the maximum TTC counts were 71 CFU/100 ml and the mean was 4 CFU/100 ml. Thermotolerant coliform counts were above the permissible standard values for nearly 40% (n = 111) of the basic water services. TTC was detected in 44 (39.64%) (n = 111) basic water services. Of these, 38 (34.23%) were operationally functional while 6 (5.41%) were not functional. Approximately one third of the basic water services sampled, deemed “functional” by international standards, do not provide *potable* water due to thermotolerant coliform (TTC) levels.

**Conclusion:**

Our findings from the Amhara and Afar regions of Ethiopia demonstrate that water quality parameters are not currently considered in classifying basic water services. This suggests that international efforts to address SDG 6 should incorporate water quality as a key parameter to better track international progress toward “clean water and sanitation” efforts. We discuss two potential pathways for stronger inclusion of water quality parameters in international definitions: (1) to mandate water quality within “functional” and “non-functional” definitions or (2) to add a ladder rung titled “safe basic water services” to the international drinking water ladder. Our findings from Ethiopia suggest that additional research should be undertaken in development contexts to assess whether or not “functional” basic water services provide safe drinking water to users.

## Introduction

The Sustainable Development Goals (SDGs) coordinate international efforts toward improving the lives of people around the globe. SDG 6 broadly outlines “clean water and sanitation” while target 6.1 specifies: “By 2030, achieve universal and equitable access to safe and affordable drinking water for all” [1]. Yet achieving safe drinking water in developing countries and resource-poor environments requires sustained effort. In contribution, we demonstrate that current international standards around improved water have not properly specified standards around water quality. Without a stronger incorporation of water quality parameters, international targets risk focusing in improved basic water services means water free from contamination that merely supply users with non-potable, and contaminated water that would end up with non-functional. Water contaminated with thermotolerant coliforms (TTC) will not achieve the SDGs in the lives of people around the world. Using the case of water in Ethiopia, this paper documents the existence of diarrheal bacteria (TTC) in water services currently deemed functional by international standards. We then utilize qualitative methods to contextualize water service management by local citizen committees, called WASHCos (Water, Sanitation, and Hygiene Committees). While our data is specific to Ethiopia, we find 34% of basic water services currently classified as functional, do not offer “safe drinking water” as SDG 6.1 states as a global goal. This is supported by other study conducted in Ethiopia that resulted the key factors for improved water services functionality were improving water quality by 61% of the respondents [2]. Further, study conducted in developing countries recommends to harmonize and standardize water functionality monitoring, it is must to address water quality [3]. In a study conducted in Malawi, the water in samples (32%) conformed not met to the drinking water standard for the Malawi Government (50 CFU/100 ml) for TTC (*Escherichia coli)* and most samples (87%) did not meet the WHO drinking water standard of zero CFU/100 ml) of *E*. *coli*. Thus, the water services that are working well tend to have the lowest *E*. *coli* contamination levels [4]. This demonstrates the importance of including a water quality parameter, such as the presence of TTC, to measures basic water functionality. Basic water services are the main source of drinking water for communities around the globe, therefore understanding water quality is an essential component to achieving universal access to safe and clean drinking water [5].

International standards measure and classify water services and water quality in various ways. At the highest level basic, water services are deemed improved or unimproved and water services are classified as functional or non-functional [5, 6]. The unimproved sources include drinking from unprotected dug wells or springs or directly from the source, such as a river, lake, pond or irrigation canal/channel. This article is focused on improved water services which include: rural piped system (RPS) from a spring, borehole or shallow wells, hand-dug wells with hand pumps (HDW), rainwater harvesting (RWH) structures, constructed on-spot springs, reservoirs, river intakes (RI), and slow sand filters (SSF) [7]. Improved water services are then understood as water services (accounting for both the source and water delivery systems), classified as safely managed, basic, or limited with set parameters for each (Table 1). Improved water services are classified as functional (fully operational system at time of survey) or partial or fully non-functional (fails to operate at time of survey) [6].

**Table 1:** SDG definitions for the classification of improved water services.

**Safely managed services**	1. Source is accessible on premises **and**
	2. Water is available on demand **and**
	3. Water is free from contamination
**Basic water services**	If source does not meet any **one** of the three criteria **and** a round trip to collect water takes 30 minutes or less including queuing.
**Limited water services**	If source does not meet any **one** of the three criteria **and** a round trip to collect water exceeds 30 minutes.

However, as is seen in Table 1, water quality (measures that assess contamination) is a mandatory criterion for safely managed services yet becomes an *optional criterion* in the basic and limited water services definitions. In other words, a basic water service may be classified as “functional” even if the water is not, in practice, “safe drinking water”—the goal of SDG 6.1. The flipside of this is that water services classified as “non-functional” do not incorporate water quality. Water quality parameters include testing samples across three dimensions: biological (total coliforms (TC) and TTC in colony forming unit (CFU) per 100 ml of water samples), physical (turbidity in nephlometeric turbidity unit (NTU)) and chemical (‘pH’ and free residual chlorine (FRC) in milligrams (mg) per liter (lit) of water samples). The SDGs classify safe drinking water based upon the updated World Health Organization (WHO)/United Nations Children’s Fund (UNICEF) joint monitoring programme (JMP) ladders of safe drinking water [1]. This classification does not, we argue, properly account for “safe” drinking water in basic water services. The inclusion of a fecal indicator bacteria (FIB) as part of water quality in the commonly-accepted definition of basic water [1] is a gap that obscures the risk of diarrheal diseases from water services classified as both improved and functional. Given the morbidity and mortality caused by diarrheal disease and the disproportionate burden placed onto under-five children and women [8, 9], a stronger incorporate of water quality parameters is essential to driving investment in water services that produces meaningful enhancements in the lives of Ethiopians and developing country citizens more broadly.

Functioning water services are fundamental to social and economic development: improving the quality of health and educational achievement by reducing the morbidity and mortality, malnutrition, stunting rates of people in community and improving livelihoods [8, 9]. Global data on water services show that 844 million people still lack basic drinking water. Two hundred sixty-three (263) million people spent over 30 minutes per round trip to collect water from an improved sources due to scarce drinking water services [1]. One hundred fifty nine (159) million people, of which 58% are in sub-Saharan Africa, continue to fetch drinking water directly from unimproved sources such as unprotected wells and springs or surface water services [1]. Indeed, enhancing the existing basic water services to meet water quality standards is a strategic development area for increased investment.

Despite efforts to increase the safe service of water with an ultimate outcome of improving the health and nutritional status of the community [10, 11] in food insecure rural areas of Ethiopia, with special attention to Amhara and Afar regional states, malnutrition rates in these two regions exceed the national average (40%) [12]. In Ethiopia, lack of access to water supply causes around half of all health complications from undernutrition [8]. Since 1990, joint efforts by the Ethiopian government and numerous non-governmental organizations (NGOs) have expanded services to water, sanitation and hygiene (WASH) through the construction of water infrastructure in Amhara and Afar of Ethiopia [1, 7]. While much focus has been given for the construction of new water services, there is inadequate or no data on existing water services that, after construction, require on-going maintenance. Once a water service is constructed and therefore “improved,” it may then be considered as safely-managed, basic, or limited. The negative impact of water quality apart from physical drying or low yielding as well as weak operation and maintenance of water on the functionality of basic water services were reported in studies [2, 3, 13]. In the case of rural Ethiopia, partial functionality and non-functionality of basic water services were reported as prevalent challenges [13]. Ethiopia is not alone in its developmental struggle for clean water accessible from improved sources.

In Ethiopia, the proportion of people using a **safely managed** water service (accessible on premises, available when needed, and free from contamination) is only 11% (4% rural and 38% urban) [1]. This data indicates a significant gap between urban and rural areas residents, while demonstrating that even urban living in Ethiopia does not equate access to clean water. To understand this at a nation-wide scale, out of the sixteen small-to-medium towns in Ethiopia, only 2 towns provide water that meets the Ethiopian government’s Growth and Transformation Plan I standard [14] which requires water be free from contamination. Moreover, **basic water services** meaning water from an improved source does not meet any one of accessible on premises, available when needed and free from contamination, but a round trip to collect water takes 30 minutes or less including queuing is the next category in the water ladder. Therefore, where water quality may or may not meet the joint monitoring program (JMP) definition of basic water services standards, in Ethiopia, the national drinking water coverage of basic water services is 39% (30% rural and 77% urban) [1]. In Ethiopia, 12% of the national population still relies on untreated surface water [15], and within the rural population 47.2% use surface water and 28.1% use spring water services, meaning that nearly 75% of the rural population uses water from unimproved sources [16].

Rural Ethiopian communities have struggled to first gain access to basic water services and then maintain their functionality. In rural Ethiopia in 2013, a total of 92,588 water services were inventoried by Ministry of Water, Irrigation and Electricity of Ethiopia. As can be seen in the inventory data, the overwhelming majority, 78.4%of rural Ethiopians get water from basic water services. The types of the basic water services include: hand dug wells with hand pumps, springs without distribution networks, shallow wells with hand pumps and rope pumps [17]. As in many other countries, in addition to limited *access* to basic water services, non-functionality of newly built or existing basic water services is a major problem in Ethiopia [13]. One study, based on 12 countries across African and Asian continents, found that 28.7% were non-functional or partially functional [18]. In the same study, non-functionality of water systems in Sub-Saharan African countries (focused on Ethiopia, Ghana, Kenya, Rwanda, Uganda, and Zambia), were 24.8%, followed by 21.3% in Asia (India) and 7.3% in Latin America & the Caribbean (El Salvador, Guatemala, Haiti, Mexico, Nicaragua) [18]. According to Ministry of Water Irrigation and Electricity inventory report, the national non-functionality rate for Ethiopia is estimated to be 26% with the highest rate of non-functionality (34%) in the Afar region [17]. In Ethiopia, the non-functionality rate of water services was found to be 20–30% nationally and up to 50% for rural Ethiopia [16]. Additionally, in a study across four Ethiopian regional states that are considered to have stronger infrastructure than the remaining five regions, 20% of water services were found to be non-functional [2].

Studies find different reasons for the non-functionality of water services in Ethiopia. One study [19] attributed that 50.8% (n = 102) of users fail to pay water service fees, which allow for consistent operation and minor maintenance as needed. Another study documented that 53% of water services lack a budget source, 40% had no trained caretakers, and only 7% have limited access to spare parts [20]. Ethiopia utilizes similar water management structures as seen elsewhere, such as Ghana and Uganda [21, 22]: basic water services in rural areas are managed by volunteer citizen Water, Sanitation and Hygiene Committees, popularly called “WASHCos.” Each WASHCo consists of 7 or more individuals, with government mandates to include 4 women and 3 men from the community. WASHCo members are trained across a variety of dimensions by both governmental and non-governmental actors [23]. In general, their training aligns with international standards, which typically recognize 5 dimensions that contribute to strong water service management by local citizens. These 5 dimensions include: technical (e.g. water technicians, spare parts), institutional (e.g. meeting frequencies, capacities of members, promotion to potential service users), social (e.g. gender composition, working relationships), environmental (e.g. drought, flooding) and financial (e.g. ability to collect water fees, cost of operation and maintenance). Poor performance across these 5 dimensions is thought to contribute negatively to water service functionality. Despite the limitations of WASHCos, the presence of a WASHCo improves the ongoing functionality and water services, and WASHCos perform better than non-community managed types such as local government and private entities [23]. Lastly, community perceptions of water quality enhance use of basic water services, which further helps drive functionality through perceiving water quality [24], timely paying of fees, and speeding up repair times [2].

Consequently, the aim of this assessment was to assess the status of basic water services functionality with respect to water quality and WASHCo management in Amhara and Afar regions of Ethiopia. The study takes into account water quality, as measured by the presence of fecal indicator bacteria (FIB) as one factor of functionality in addition to water service managerial factors such as technical, institutional, social, environmental and financial aspects. The study also identifies potential gaps to be addressed for improvement. Our data demonstrate that too many factors have been confounded into water services that are classified as “basic.” In contribution we suggest that the JMP ladder either incorporate water quality into the criteria for basic water services or add an additional rung to track and develop more nuanced targeting around basic water services that provide “safe drinking water.” The study provides evidence in support of the Ethiopian government’s commitment to reducing the non-functionality rate (NFR) to 7% as stipulated in the One WASH National Program II (OWNP II), which complements and contributes to the Growth and Transformation Plan II (GTP II) [25].

Our findings suggest that water quality is an important, yet currently under-researched aspect of “safe drinking water.” By incorporating water quality parameters into basic water services, investment could be better driven to making basic water services potable rather than investing first in infrastructure development. Given the morbidity and mortality caused by diarrheal disease [26, 27], international investments should invest in functionality *and* water quality. Water quality as a metric should remain a consistent priority during the planning, implementation, maintenance, and monitoring and evaluation of water services. The findings of this study may be of use to water scholars, global development organizations, national policymakers, as well as grass-roots level implementers.

## Methods and analysis

In order to understand the functionality of basic water services, this mixed methods study collected data from 22 food-insecure rural districts, known locally as woredas, selected from two regions—Amhara and Afar. In the Ethiopian context, the country is divided into regions, then zones, then woredas, and, finally the smallest unit named kebele. In the selected woredas/districts, malnutrition rates exceed the national average of 40% [12, 28, 29]. The 111 basic water services sampled were then tested for the required priority water quality parameters such as biological: total coliforms (TC) and thermotolerant coliforms (TTC), physical: turbidity and chemical: ’pH’ and free residual chlorine (FRC). Data collection was conducted from January to June 2019: the driest peak season in the area. This season was selected for the reason that the factors related to environment and climate change would be greatest. *E*. *coli* is higher in the rainy season than the dry season in Ethiopia [30]. Therefore, by collecting data during the peak dry season, we expect our results to provide the most conservative estimations regarding the role of water quality in water service non-functionality.

### Sample size

**Fig 1:**
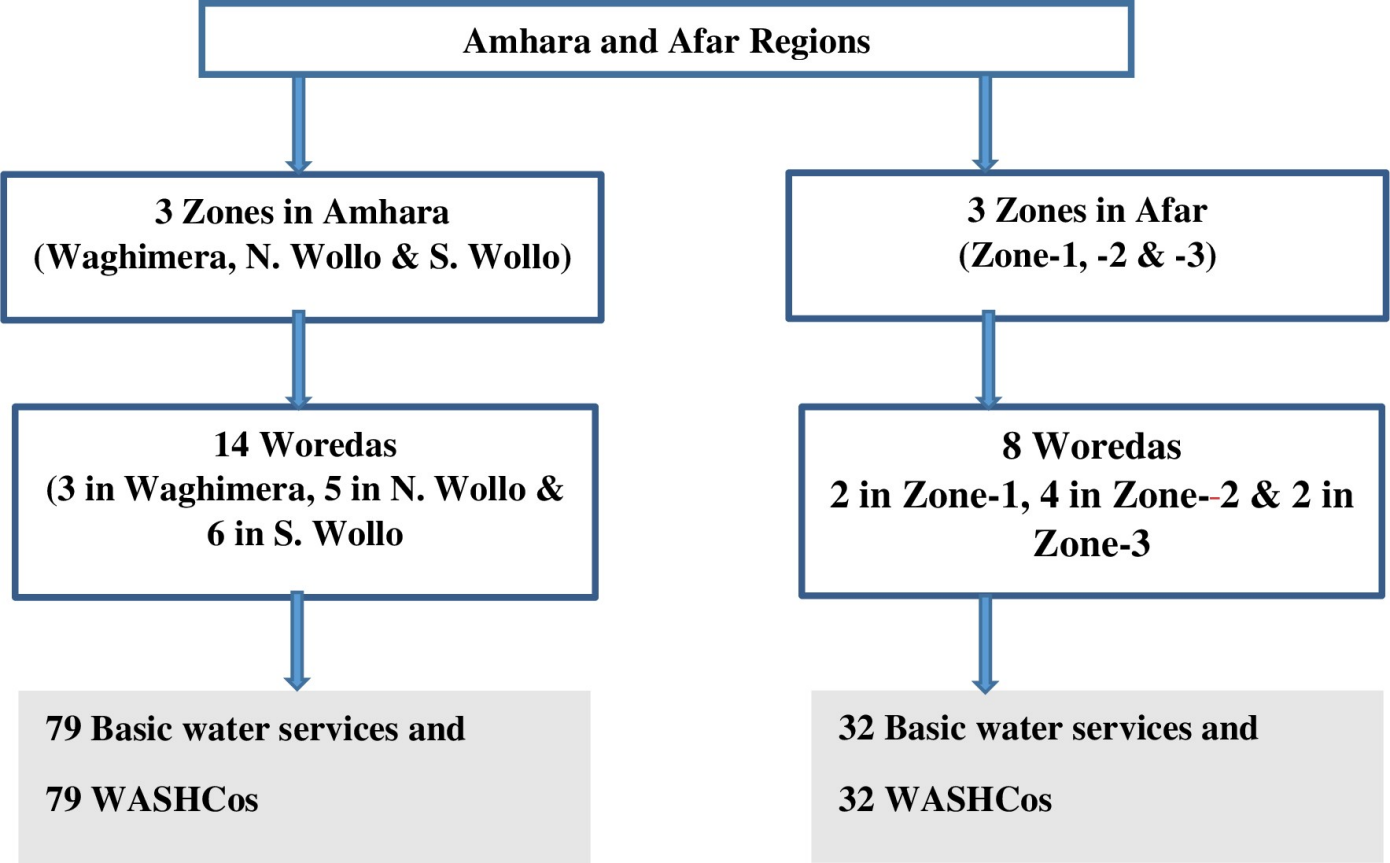
Sampling structure of selected basic water services and associated WASHCos.

Amhara and Afar regions were purposively selected due to their high levels of malnutrition and food-insecurity, and then within each region three zones were randomly selected. Within the selected zones, using government lists of food insecure woredas/districts, a total of 22 woredas/districts were selected (Fig 1 and Table 2).

**Table 2:** Summary of sample in comparison to total basic water services population in study area.

Region	Zone	Total basic water services in Zone n =	Total basic water services in selected Woredas n = (*n = selected Woredas*)	Sampled basic water services (n =)	Sample as a percent of Woreda total
Amhara	Waghimera	120	82 (*3*)	14	17.07
	North Wollo	920	327 (*5*)	33	10.09
	South Wollo	963	346 (*6*)	32	9.25
	**Sub-total**	**2003**	**755 (*14*)**	**79**	**10.46**
Afar	Zone-1	37	22 (*2*)	3	13.64
	Zone-2	46	31 (*4*)	19	61.29
	Zone-3	54	39 (*2*)	10	25.64
	**Sub-total**	**137**	**92 (*8*)**	**32**	**34.78**
	**TOTAL**	**2140**	**847 (*22*)**	**111**	**13.11**

Following the selection of targeted woredas/districts, a total of 111 (79 in Amhara and 32 in Afar) basic water services and respective WASHCos were selected randomly from the water services inventory lists of the Zone Water Offices. This represents approximately 13.1% of the total number of basic water services of the water services found in the 22 woredas/districts and their respective WASHCos (Table 2).

### Methods

This mixed methods research focused on basic water services as the unit of analysis. Assessing each water service identified by the sampling technique described above, we then created an in-depth understanding of each water service using mixed methods. This study includes five data sources taken from each sampled water service and corresponding WASHCo: geocoded location data for the water service, water samples taken, visual and physical inspection of water service with an observation checklist, interviews of 2–3 WASHCo members using a structured questionnaire, and focus group discussions (FGDs) with the remaining 4–5 WASHCo members using a checklist (S1 File). In addition to the above primary data collection, the research team analyzed individual WASHCo records such as meeting minutes, bank or microfinance books, water fee collection receipts and water users lists that were kept about their basic water services.

First, geocoded data to geo-locate the water services were collected using instruments GPS-garmin-62 with an accuracy to three meters (3m) precision. Second, water samples of 300 ml were taken from all sites for lab analysis, irrespective of the functionality of the basic water services (either from the sources or reservoirs or containers or public water points/tap stands). Water quality was assessed from water samples across biological, physical, and chemical parameter properties that include the following tests: **bacteriological**: total coliforms (TC) and thermotolerant coliform (TTC) using membrane filter (MF) technique of International Standard Organization (ISO): 9308–1:2014 detection and enumeration of TC and TTC bacteria [31–33] were tested. Membrane Lauryl Sulphate Broth (MLSB)-AVONCHEM-ACM-1820-O media was used for the bacteriological- TC and TTC detection. Total Coliforms were detected by culturing at 37 ^0^C for 24 hours. Thermotolerant Coliforms were detected by culturing at 44 ^0^C for 24 hours. The growths of yellow colonies in each plate on the filter papers were directly counted in each quadrant. The results in numbers and in CFU/100 ml registered in a laboratory. Physical, turbidity (NTU), and chemical, ’pH’ and free residual chlorine (FRC) in mg/lit, were tested using photometer 7100 of Palintest (manufactured by Wagtech in United Kingdom) with preprogrammed test calibrations. Water quality test protocol is found in this link: https://dx.doi.org/10.17504/protocols.io.bpc7mizn. Third, direct visual observation of water services was conducted. This included: functionality factors in terms of technical aspects; service type, technology type, the status of water services, training, experience of operation and management (O&M), access to spare parts, water treatment/chlorination collected.

Qualitative methods were used to collect data from WASHCo members. The WASHCo members were randomly assigned to either interviews or FGDs, however women were purposively sampled for inclusion to ensure the presence of one woman in both the interview and FGD. The questions in the interview and FGD questionnaire are nearly similar (S1 File). In this manner, data was verified across observations, interviews, and FGDs. This triangulation is important to improve data quality since many of the WASHCo members have low literacy and numeracy and may struggle to recall particular details about the water service. Both interviews and focus group discussions were given by research assistants hired for each local area. Interviews and focus group discussions were conducted by research assistants who hold a bachelor’s degree and have a background in water and sanitation.

### Data collection and analysis

The study data was gathered using the partially modified and piloted questionnaire from the standard water functionality survey questionnaire, FGD checklist and lab analysis by obtaining informed verbal consent from each of the participants of WASHCo members.

Depending on the nature of variables, an analysis tool was developed using IBM SPSS statistics software version-20. Before the analysis, data were cleaned and validated. Descriptive statistics of frequency, percent, mean, standard deviations, normal tables, cross-tabulations and graphs were used. A one tailed *t*-test at 95% CI was used to evaluate whether the mean for microbial parameters (TC and TTC counts), physical parameter (turbidity) and chemical parameters (‘pH’ and residual chlorine) in the water were significantly different from the WHO and Ethiopian quality standards.

The protocol for this study was approved by the College of Natural and Computational Science Institution (CNS-IRB), Addis Ababa University Review Board (CNSDO/729/10/2018) dated July 24, 2018. Permission was obtained from the community members orally after explaining the study objectives and how they were selected for this study because of lack of reading and writing by included cases. Confidentiality of information was respected.

## Results and discussions

### Location and type of water services

A total of 111 (Afar: n = 32, 28.8% and Amhara: n = 79, 71.2%) basic water services and their respective WASHCos found in six zonal administrations that cover 22 woredas/districts were included in this study. In Amhara, the water services included were located in North Wollo 33 (10.09%), South Wollo 32 (9.25%) and Waghimera 14 (17.07%) of the total basic water services found in the 14 woredas of Amhara. Likewise, the Afar samples were 19 constituting 61.29% in Zone-2, followed by 10 (25.64%) in Zone-3 and 3 (13.64%) in Zone-1 of the total basic water services found in 8 woredas of Afar see (Table 2) above.

**Fig 2:**
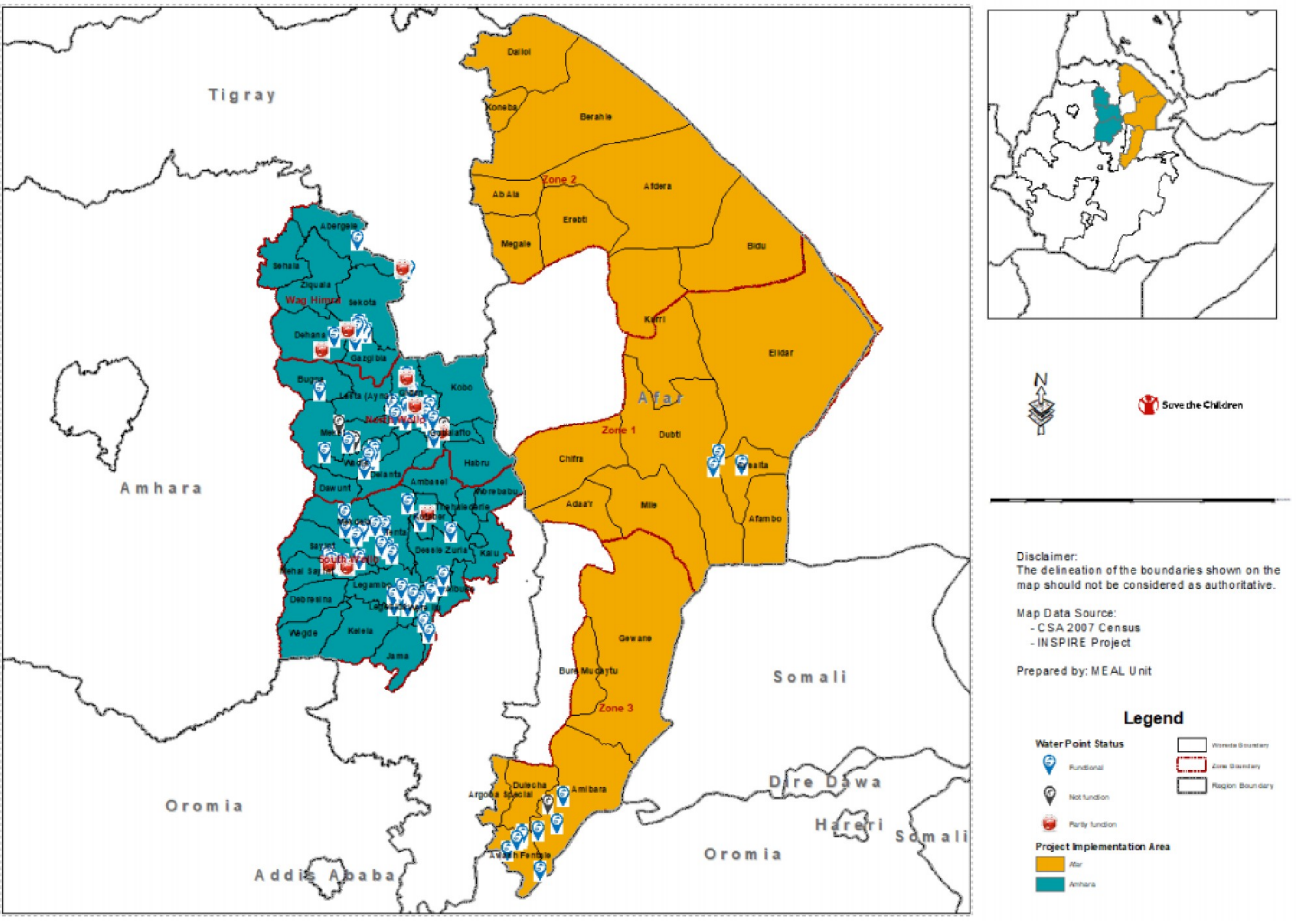
Location map of the study sites, Amhara and Afar, Ethiopia (Save the Children GIS center is used to develop this map).

The location map of the 111 basic water services included in this study was geo-referenced. Global positioning system (GPS) reading; X (longitude), Y (latitude)-coordinates and Z (altitude) data were taken. However, some information was difficult to include in the data from Afar (shown in Fig 2). The water points were located at a minimum altitude of negative 91 meters below sea level (BSL) in Afar with arid climatic conditions and a maximum altitude of 3,402 meters above sea level (ASL) in Amhara characterized by cold fertile climatic conditions. The mean altitude reading of the locations of the basic water services was 2,076.62 meters above sea level and standard deviation of 953.338 meters (Fig 2). The type of the basic water services in the Amhara highlands might vary from that of the lowlands in Afar. This would have brought different factors that contribute to the functionality of water services and the water contamination itself as functionality factor. In a review of case study conducted in Malaysia using 68 studies, it was confirmed that factors such as soil erosion, landslides and agricultural activities associated with land use change have significantly influenced the water quality in the highland areas [34] similar to the Amhara region. This might not be the case for Afar region, with lowland areas where the life of the community was dependent on livestock in pastoralist area.

### Water services: Technical findings for functionality

The basic water service technology types included in this study were rural piped system (RPS) using 42 spring sources (37.8%), 25 hand-dug wells with hand pumps (HDW) (22.5%), 22 RPS from a motorized borehole or shallow wells sources (19.8%), 9 rainwater harvesting (RWH) structures (8.1%) and others such as on-spot springs, reservoirs, river intake (RI) and slow sand filters (SSF) jointly account for 13 basic water services (11.8%). It was found out that treatment of water is largely done by WASHCos (n = 43, 38.7%) and Woreda Water Offices (WWO) (n = 38, 34.2%) while very few sources (n = 2, 0.9%) were reported to be treated by Zonal Water Offices (ZWO) and others such as tap attendant (one source by each). The time interval of water chlorination is often quarterly (n = 46, 41.5%), several others yearly (n = 21, 18.9%), and every six months (n = 13, 11.7%) and the remaining few (n = 3, 2.7%) monthly. Unfortunately, as to interviewed WASHCos, a quarter of the water services (n = 28, 25.2%) sampled in this study were disinfected totally by any responsible body including the WASHCos who are largely responsible for the disinfection of the water services (Table 3).

**Table 3:** Results of the technical variables.

Variables		Size	Percent
Water service types	On spot springs	6	5.4
	RPS-from gravity springs	42	37.8
	Motorized shallow or bore wells	22	19.8
	Hand-dug wells	25	22.5
	Rainwater harvestings	9	8.1
	Water reservoirs	4	3.6
	River intake	1	0.9
	Slow sand filtrations	2	1.8
Status of water services	Functional	87	78.4
	Not functional	24	21.6
Responsible body to treat water	WASHCos	43	38.7
	Woreda Water Office	38	34.2
	Zone Water Office	1	0.9
	Other-tap attendant	1	0.9
Time interval to treat water by WASHCos/others	Monthly	3	2.7
	Quarterly	46	41.5
	Every six months	13	11.7
	Yearly	21	18.9
	Not disinfected	28	25.2

From our sample of 111 water services, 24 sources (21.6%) were categorized as non-functional from technical and management aspect by interviews and FGDs with WASHCos, and water services visual observation. The reasons for non-functionality of basic water services listed in decreasing order of importance, excluding water quality, were poor management (n = 19, 17.1%), unavailability of spare parts (n = 17, 15.3%), lack of maintenance tools (n = 12, 10.8%) and environmental reasons (n = 10, 9.0%). Indeed, lack of skilled technicians and shortage of money were also barriers to functionality (Table 4).

**Table 4:** Reasons attributed to the non-functional water services other than water quality.

Reasons for non-functionality	Response frequency (%)	
	Yes	No
Poor management	19 (17.1)	5 (4.5)
Unavailability of spare parts	17 (15.3)	7 (6.3)
Lack of tools	12 (10.8)	12 (10.8
Environmental reasons	10 (9.0)	14 (12.6)
Shortage of money	7 (6.3)	17 (15.3)
Lack of skilled technicians	6 (5.4)	18 (16.2)

### Water services: Water quality

At present, water quality is not a parameter considered under the classification of “functional improved basic water.” Water quality is a neglected topic in global debates and quality is not given due attention as it should be [1, 35] as far as basic water services is concerned. Preserving the quality of basic water services, which the majority of the people in developing countries have access to, is important for better drinking-water supply use and decreasing morbidity. Water quality lab analysis were conducted for all basic water services using lab protocol (S2 File). Water samples for lab analysis were taken from the sources for functional basic water services and for the non-functional 24 (21.62%) of the water services, the samples for lab analysis were taken from either at the sources or storages or in the pipelines. In the lab analysis, measured in colony forming counts per 100 milliliters (ml) of water samples for **bacteriological tests** of total coliforms (TC) were detected from 46 (41.44%) taken from basic water services. Of which 40 (36.03%) of the basic water services are functional and the remaining 6 (5.41%) were not functional.

The other test conducted from the priority bacteriological tests is the thermotolerant coliforms (TTC). The presence of TTC serves as an indicator of water contamination by human or animal fecal matters. TTC was detected in 44 basic water services of (39.64%) of which 38 (34.23%) are operationally “functional” and 6 (5.41%) are not functional (Table 5). Therefore, TC for about 41% and TTC for nearly 40% of the water services were above the permissible standard values, which is 0 CFU/100 ml.

**Table 5:** Water quality parameters versus services functionality in Amhara and Afar regions.

Parameters	Classifications	Water services		
		Functional	Non-functional	Total n (%)
**Total Coliform**	Meets standard of 0 CFU/100mL	47 (42.34)	18 (16.22)	**65 (58.56)**
	Above standard of 0 CFU/100mL	40 (36.03)	6 (5.41)	**46 (41.44)**
**Thermotolerant Coliform**	Meets standard of 0 CFU/100mL	49 (44.14)	18 (16.22)	**67 (60.36)**
	Above standard of 0 CFU/100mL	38 (34.23)	6 (5.41)	**44 (39.64)**
**’pH’**	Below the standard– 6.5	1 (0.90)	1 (0.90)	**2 (1.80)**
	Meets standard of– 6.5–8.5*	47 (42.34)	17 (15.32)	**64 (57.66)**
	Above the standard– 8.5	39 (35.13)	6 (5.41)	**45 (40.54)**
**Turbidity**	Meets standards of less than 5 NTU	83 (74.78)	24 (21.62)	**107 (96.40)**
	Above standard greater than 5 NTU	4 (3.60)	0 (0.0)	**4 (3.60)**
**Residual Chlorine**	Below the standard– 0.2 mg/lit	86 (77.48)	23 (20.72)	**109 (98.20)**
	Meets standard– 0.2–0.5 mg/lit*	1 (0.90)	0 (0.0)	**1 (0.90) **
	Above the standard– 0.5 mg/lit	0 (0.0)	1 (0.90)	**1 (0.90)**

* WHO drinking water quality guideline recommended standard.

**Fig 3:**
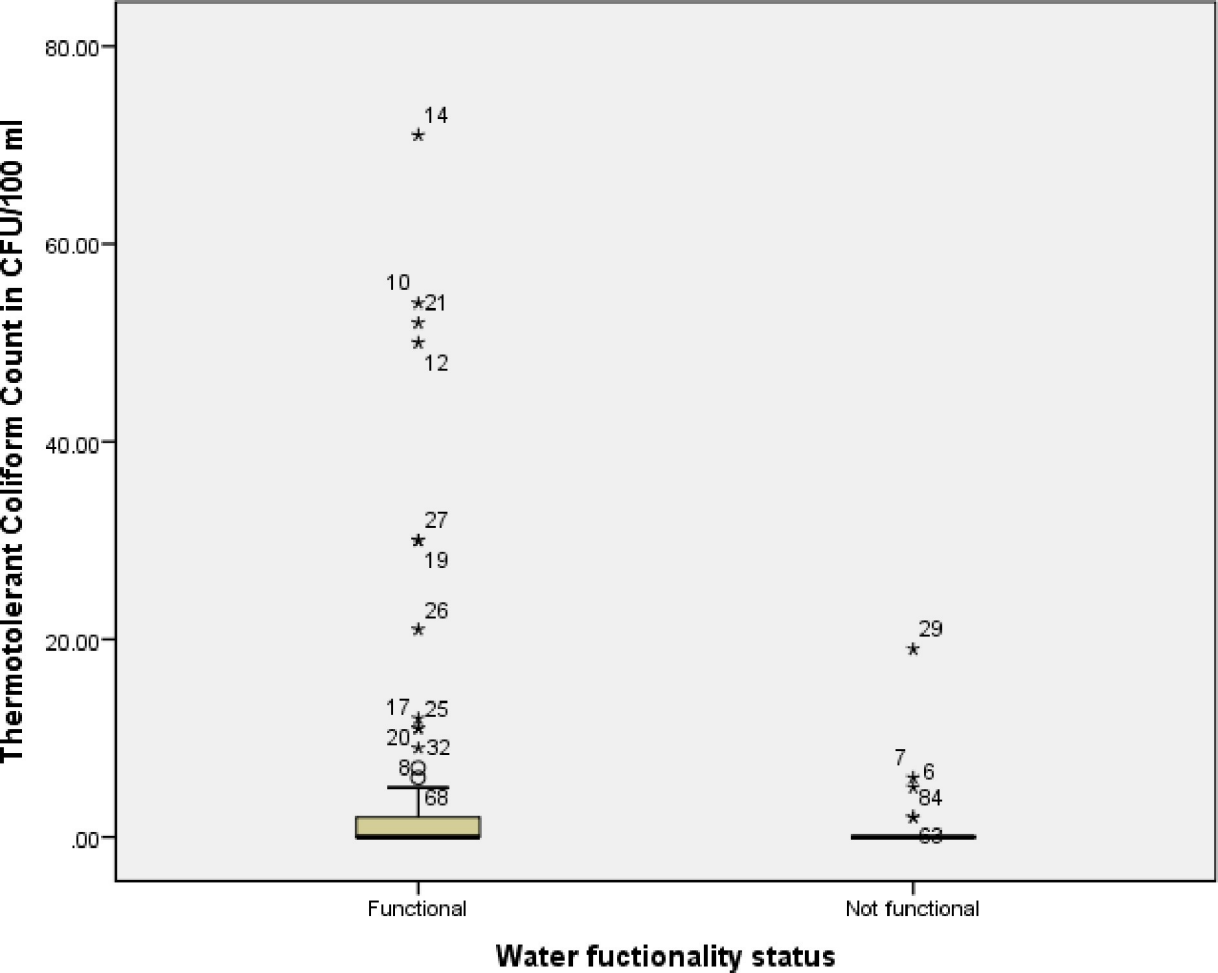
Water functionality status compared to the detection of TTC in CFU/100 ml.

To compare the dispersion of the result of the thermotolerant coliform counts detected in functional and non-functional basic water services we used box plot. Thus, comparing the thermotolerant coliform in functional and non-functional basic water services, there is greater variability in detected thermotolerant coliform counts in functional basic water services than non-functional basic water services as shown in the box plot (Fig 3). In other words, the data set is highly dispersed in functional basic water services than non-functional basic water services.

The maximum total coliform (TC) counts was 111 CFU/100 ml and the mean was 11 CFU/100 ml. The maximum TTC counts were 71 CFU/100 ml and the mean was 4 CFU/100 ml. As to priority chemical tests, the ’pH’ of 47 basic water services (41.34%) were either below or above the allowable limits which is in the range of 6.5–7.5. In addition, FRC result shows that 109 basic water services (98.20%) were below the allowable ranges– 0.2–0.5 mg/lit. The mean of free residual chlorines and ’pH’ were 0.04 mg/lit and 7.4 respectively. The mean ’pH’ result was within the range of permissible values of the standard, while the mean of FRC is below the permissible values of the standard-0.2–0.5 mg/lit in basic water services. The low level of the FRC could result in reduced killing of pathogenic microorganisms. This creates a higher risk of contamination of the water services by human and or animal wastes. This high-level contamination was confirmed by the detection of the TC and TTC in the water samples brought from different water services. 107 of the basic water services (96.40%) showed turbidity values within the acceptable value of the drinking water quality guideline of World Health Organization (WHO) (less than 5 NTU) (Table 6). Therefore, the lab test result of the five (TC, TTC, ‘pH’, turbidity and residual chlorine) primary water quality parameters minimum, mean, maximum detection level was computed with the WHO and Ethiopian standard (Table 6).

**Table 6:** Summary of water quality results of basic water services sample (n = 111) in comparison to WHO and Ethiopian standards.

		Water Quality Parameters				
		TC Count CFU/100 ml	TTC Count CFU/100 ml	‘pH’	Turbidity (NTU)	Residual chlorine (mg/lit)
WHO & Ethiopian Standards*		**0**	**0**	**6.5–8.5**	**< 5**	**0.2–0.5**
**Sample**	**Minimum**	0	0	6	0	0
	**Median**	0	0	7.48	0.75	0
	**Mean**	11	4	7.48	1.36	0.05
	**Maximum**	111	71	8.81	31	5.3
	**Std. Deviation**	23.14	11.60	0.42	3.33	0.50

*Ethiopian standard authority developed similar standard to the WHO.

By considering the TTC of which the significant public health concern in causing diarrheal diseases and morbidity specially in children under-five in developing countries like Ethiopia, this study points out the contribution of water quality for non-functionality. Accordingly, an additional 38 functional water services (34.23%) were re-categorized as non-functional because of water quality and 6 (5.41%) of already non-functional as a result of technical and management factors were also unfit for consumption because of water quality, which ended up to double non-functional.

The overall non-functionality rate of basic water services 44 (39.6%) was higher than the findings of other studies. For example, in studies of a twelve-countries in Sub-Saharan African, Latin America & Caribbean and Asian, including Ethiopia (with sample size (n = 120 for Ethiopia) in 2014, the non-functionality rate was 28.7% [18] with nearly similar socio-economic representation. In Ethiopia, 35% of rural water supply systems [2] with almost similar socio-economic condition of our study area and with lesser sample size (n = 74) than our study, the water services were not working at the time of the survey. In other study in Ethiopia, 18% of the hand pump borehole water services [36] that consider water sample size of (n = 200) were not working at the time of the survey. These differences could be due to the consideration of water quality parameter as part of functionality for water services in this study, which was not the case in the previous studies.

Report showed that 20.6% of water services were not working at the time of the survey in Grater Afram Plains region of Ghana [37] with different size of water service sample size (n = 1509) and different socio-economic situation with our study. This variation in results come up because of the percentage variations in water contamination. In fact, the difference in sample size and socio-economic consideration between the study areas might have contributed. On the other hand, the result 44 (39.6%) of non-functionality of our study was lower than the non-functionality rate of other study in Ethiopia that shows 44.4% for shallow and boreholes [19]. This difference in non-functionality was attributed to differences in water service technology types (the present study uses variety of water service technologies and the later study considers only shallow or borehole water services) within similar socio-economic conditions and nearly the same sample size (n = 102) as that of our study. This shows that if water quality indicators are included in the later study, the non-functionality rate would be much higher than our finding.

**Fig 4:**
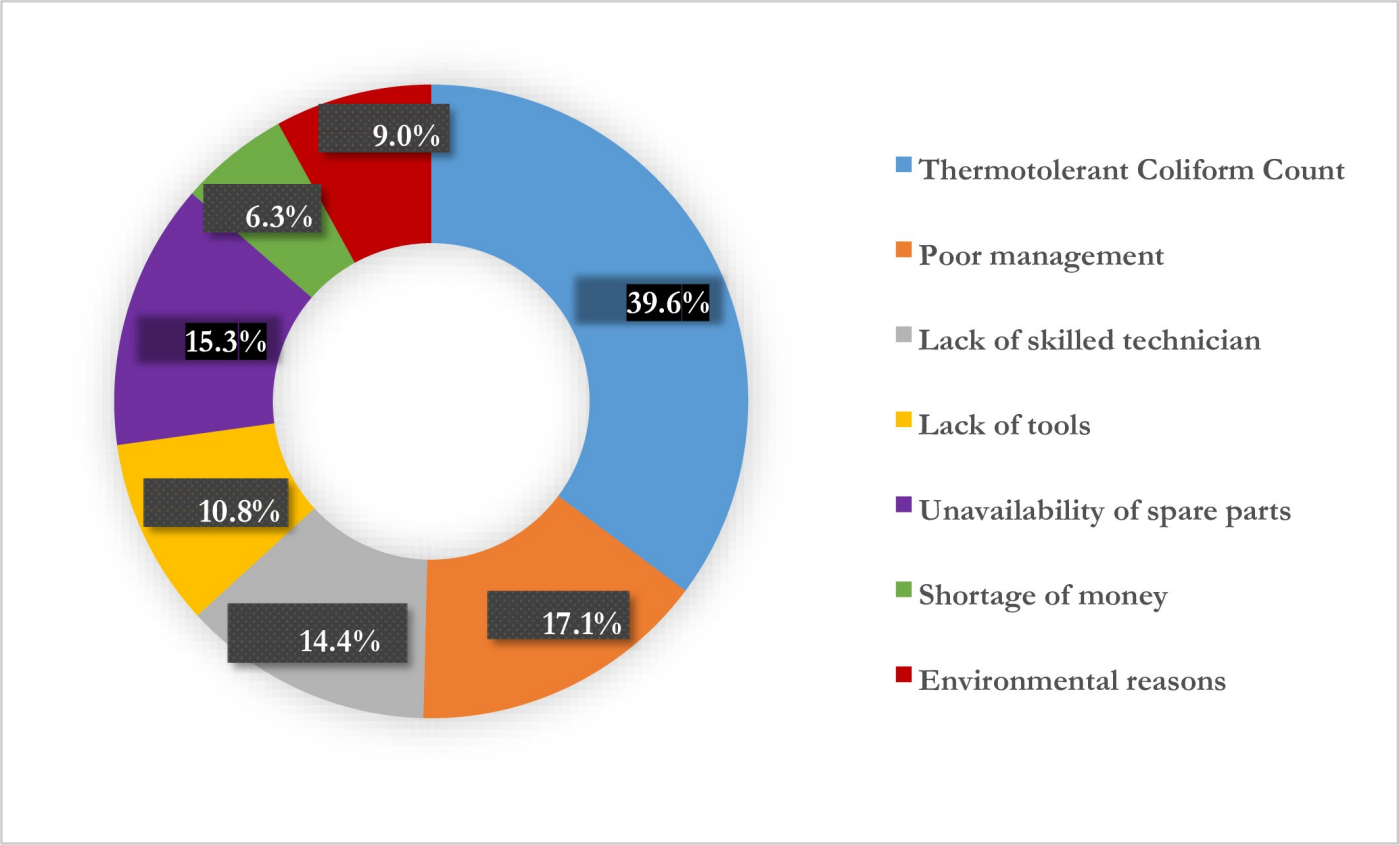
Factors resulting in basic water services non-functionality in Amhara and Afar regions, Ethiopia.

In summary, the reasons contributed for non-functionality of water services based on the laboratory analysis of water quality was **water quality-thermotolerant Coliforms** count 38 (34.23%) in operationally “functional” basic water services. Poor management 19 (17.1%), absence of skilled technicians 16 (14.4%), lack of spare parts 17 (15.3%) are chronologically identified reasons for non-functionality based on the interview and FGDs results. The findings of our study are consistent with other study [20] which showed that high non-functionality of water services was due to lack of spare parts and long distance to procure spare parts [38]. Lack of maintenance and operation tools 12 (10.8%), environmental factors 10 (9.0%) and shortage of money 7 (6.3%) were also found limiting factors in both Afar and Amhara regions. Data of the study, indeed, revealed that poor water quality brought about by contamination of the water services as seen through thermotolerant coliforms mainly contributed to the non-functionality in the study areas (Fig 4). Monitoring drinking-water supplies safety implies the quality of the water needs substantial improvement like monitoring and recording of access to functional water services [39].

**Fig 5:**
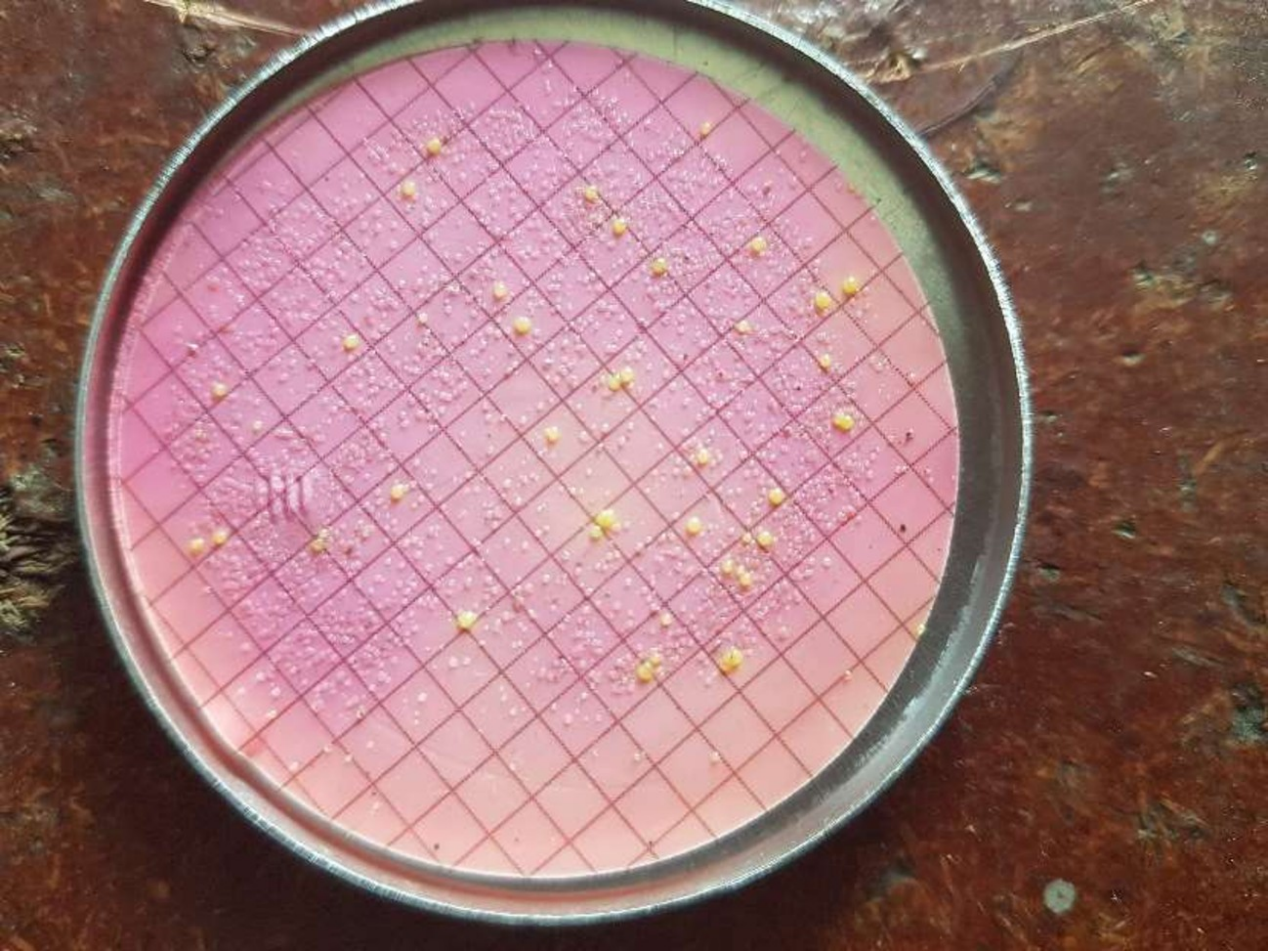
Growth of the thermotolerant microorganisms using membrane filter technique in water samples in Amhara and Afar regions of Ethiopia.

The growths of yellow colonies at 44 ^0^C for 24 hours cultured water samples using membrane filter (MF) technique in laboratory were considered that the basic water services were contaminated either with human or animal feces that prompt the water source’s status to be a non-functional category of water services. The growths of the bacterial colonies were shown in (Fig 5).

Finally, including water quality as a parameter contributes to significant reductions of non-functionality of basic water services due to poor water quality by 34.2%. Hence, improving water microbiological water quality reduces the occurrence of morbidity and mortality due to non continues flow of water and contamination of water by disease causing micro-organisms such as TTC an indicator for diarrhea causing agents of Entrobactericeae.

### Water services management: Institutional findings of WASHCos in relation to water quality

Based on the responses of WASHCos’, a significant number, 81 (73%) fully understood that the WASHCos are responsible and accountable to address water quality through water treatment/chlorination of the local water service. Twenty-three (20.7%) had partial knowledge and 7 (6.3%) did not know their duty and accountability. The significant number of WASHCos that understand their duty and accountability was also assured by the practice of WASHCos by taking over treatment of the basic water services largely (n = 43, 38.7%) (Table 3). Absence of clear role and responsibility of WASHCo membership might contribute to the loss of team cohesive work hindering the continues functioning of the water services. Almost all 110 of the WASHCos (99.1%) have reported obtaining capacity building trainings, which would contribute for those 60% of functional water services considering water quality. Overall, 108 (97.3%) WASHCos had taken training on management-related topics, 107 (96.4%) on operation and maintenance, 70 (63.1%) on sanitation and hygiene-water treatment/chlorination, 61 (55%) on child safeguarding (CSG) and 46 (41.4%) on gender. The data shows that several of them have taken two or more types of trainings (Table 7). Of the total water services and WASHCos, 20 (18.0%) were operating using tap attendants under the WASHCos structure that also contributes to the sanitation and hygiene promotion to address water treatment/chlorination. The roles of the tap attendants include among others: opening the water point on time, ensuring proper queuing by users on the basis of first come first serve, collecting water fees on the spot of the water point and handover to the cashier of the WASHCo members, cleaning the public water point areas and promotion of sanitation and hygiene. In turn, tap attendants were paid on monthly bases by the WASHCos for rendering such water services (Table 7).

**Table 7:** Results of institutional related factors in relation to water quality in Afar and Amhara regions of Ethiopia.

Variables		Count	Percent
Knowledge on duty and accountability	Yes	81	73.0
	Partially	23	20.7
	No	7	6.3
Capacity building training for WASHCos	Yes	111	100.0
	No	0	0.0
Capacity training on management	Yes	109	98.2
	No	2	1.8
Capacity training on operation & maintenance	Yes	108	97.3
	No	3	2.7
Capacity training on sanitation and hygiene-water quality	Yes	71	64.0
	No	40	36.0
Capacity training on gender	Yes	47	42.3
	No	64	57.7
Capacity training on CSG	Yes	61	55.0
	No	50	45.0
Capacity training on other topics	Yes	29	26.1
	No	82	73.9
Tap attendants for water point-hygiene promoters (water quality)	Yes	20	18.0
	No	91	82.0

Several of WASHCos 43 (38.7%) both in Afar and Amhara regions were found to access spare parts from Woreda Water Offices (WWOs), a few 19 (17.1%) from Woreda markets and some others 15 (13.5%) from Zonal markets. Even though, providing of spare parts for WASHCos by private contractors and NGOs are not a common practice, it was interesting to find out that 9 (8.1%) and 5 (4.5%) WASHCos used to get spare parts from international non-governmental organizations (INGO)/NGOs and Contractors leftover spares, respectively. Indeed, a non-negligible number 20 (18%) of WASHCos were not accessing spare part at all. Of those WASHCos who accessed spare parts, 49 (44.1%) had established controlling mechanisms on how the spare parts are being used as part of material management. Documentation practice of WASHCos was also considered in this study using WASHCos interview, FGDs and records review. As a result, beneficiaries’ lists by 86 (77.5%), saving accounts of bank by 67 (60.4%), administrative-bylaw of manuals by 57 (51.4%) and meeting minutes by 54 (48.6%) of WASHCos were documented (Table 8).

**Table 8:** Results of institutional related factors for basic water functionality in Afar and Amhara regions of Ethiopia.

Variables		Frequency	Percent
WASHCos recorded minutes	Yes	54	48.6
	No	57	51.4
Rate of meeting interval	Weekly	3	2.7
	Every two weeks	6	5.4
	Monthly	40	36.0
	Quarterly	4	3.6
	Other	1	0.9
WASHCos access to spare parts	Contractors leftover spare parts	5	4.5
	INGOs/NGOs	9	8.1
	None	20	18.0
	Woreda Market	19	17.1
	Woreda Water Office	43	38.7
	Zone Market	15	13.5
Trend for community gathering & discussion with WASHCos	Yes	74	66.7
	No	37	33.3
Accountability of WASHCos and reporting requirement	Yes	52	46.8
	No	59	53.2
Documentation of WASHCos -Financial/management manual	Yes	25	22.5
	No	86	77.5
Documentation of WASHCos- Administrative/bylaw manual	Yes	57	51.4
	No	54	48.6
Documentation of WASHCos-Minutes	Yes	54	48.6
	No	57	51.4
Documentation of WASHCos—Beneficiaries list	Yes	86	77.5
	No	25	22.5
Documentation of WASHCos-Savings account	Yes	67	60.4
	No	44	39.6

Overall, the degree of cooperation, teamwork and socialization 54 of the WASHCos (48.6%) were categorized as low and fair (Table 9). These group dynamics deserve special attention for the success of executing their roles, duties and accountabilities properly. On the other hand, water user communities resulted in a deliberate damage of water structures because of a disagreement between upper and downstream water users with respect to water sharing for domestic, animal drinking and irrigation purposes. Further, the damage of water structures for one or other reasons might contribute to water contamination due to runoff that contains human or animal wastes.

**Table 9:** The levels of cooperation of WASHCo members in Amhara and Afar regions in Ethiopia.

Variables	Response	Yes	Percent
Teamwork of WASHCos	Low	23	20.7
	Fair	31	27.9
	Good	31	27.9
	Very good	9	8.1
	Excellent	17	15.3

### Water services management: Environmental findings

Water services non-functionality was seen from environmental perspective as it is one of the factors that could contribute to low performance of water services (Table 4). Accordingly, the result shows that environmental factors that contributed to the non-functionality of water services were indicated by 10 (9%) of the study participants. Environmental factors include climate change impacts such as flood, natural land slide, thunder, water yield reduction, high windstorm and shorter rainy seasons. Flood and natural land slide might contribute for the contamination of damaged or improperly constructed basic water services [40].

Rainfall variability as a result of climate change affects many spring water services in Amhara region and rainwater dependent technology types in Afar region. This study finding was reinforced by other study findings that more than 30% of water supplies [41] were non-functional as a result of seasonal problems, which is noted also in a study conducted in urban Ethiopia [42]. Moreover, environmental indicators had statistically significant relationship with functionality in one of the most recent studies [19] conducted in rural Ethiopia. This variation could be due to inconsistent list of environmental factors considered by different researchers. For instance, the later study focused on the awareness of the community on environmental-related adaptation measures while our study emphasized the actually occurred environmental related factors affecting functionality. Relatively, the RPS from motorized boreholes or shallow wells provide consistent water services apart from its dependency on the lifting device; submersible pump and generator functionality as the buffer aquifer storage capacity remain constant. Therefore, during water supply feasibility assessment and designing, use of local climate information to reduce non-functionality of basic water services is very important.

### Water services management: Financial findings of WASHCos

The main sources of finance, water fee collection trend, mode of payments of the water fee, practice of use of available finance for operation and maintenance of water services and the overall financial management methods are variables included under the financial factors of functionality of basic water services in our study. According to the large majority of respondents (n = 97, 87.4%), the main source of finance for the WASHCos was water fees collected from users. Our result is higher than the result of a study [19] where 49.2% (n = 102) water users collect water fee in similar socio-economic status to our study area, rural areas of Ethiopia for day-to-day operation and minor maintenance. This high level of water fee payment observed in this study might be explained by the variations in motivation and capacity building works provided for WASHCos. Based on our finding, the minimum and maximum amount of money available in the bank or microfinance per WASHCos was nil and 370,000.00 ETB or (USD$0.00–11,200), respectively.

Overall, the total sum of the 111 WASHCo’s finance was 742,275.00 ETB or (USD$22,490.00) with a mean value of 6,691.66 ETB or (USD$203.00). The mode of payment of water fee was predominantly 61 (55%) by WASHCos on a monthly basis. The study reveals that nearly one-third of WASHCos and water services (n = 35, 31.5%), didn’t start water fee collections. This was however lower than data of another study that reported 53% (n = 357) did not pay water fees [20]. In fact, the better fee collection attained by the participants of the present study might be attributed to strong motivation and capacity building efforts of the WASHCo’s. To overcome the ever-increasing basic water operation and maintenance costs, there is a need to encourage water services that lack collections to commence fee payments. Likewise, capacity building that included water quality-water treatment/chlorination, hygiene promotion and other incentive should continue for an improved maintenance of those, which are already practicing fee collection.

The study showed that among those who used to collect water fees, the maximum amount is 50.00 ETB (USD$1.51) per household (HH) per month with a mean value of 4.70 ETB or (USD$0.14) and standard deviation of 8.00 ETB or (USD$0.24). This finding is partly similar to the result of a study [23] that reported 3.2 ETB (USD$0.10) per household per month with standard deviation of 1.8 ETB (USD$0.06). Regarding the water fee collections within the custody of committee members 32 (28.8%) WASHCos were found to hold the allowable limits (<2,000.00ETB) for day-to-day O&M as per the agreed bylaw. Some WASHCos has never spent on O&M whereas, some other WASHCos had an expenditure of a maximum of 100,000.00 ETB or (USD$3,030.00) for O&M. The total sum of expenditure used for O&M by WASHCos were 473,808.00 ETB or (USD$14,360.00) with a mean amount of 4,307.35 ETB or (USD$130.00) and standard deviation of 13,977.75 ETB or (USD$425.00). Nine (8.1%) of WASHCos were used cash amount up to 2,000.00 ETB or (USD$60.00).

Moreover, there were total of 17 WASHCos (15.3%), which used cash amounting to greater than 2,000.00 ETB or (>USD$60.00) (Table 10). Effective mechanism of implementation of the basic water services tariff, supported with live business plan depending on the nature and technology types and the capital incurred during construction of the technologies, as well as including water quality improving activities is fundamental for functional basic water services and its benefit over time.

**Table 10:** Results of financial sources and its management in Amhara and Afar regions of Ethiopia.

Financial factors		Count	Percent
Sources of finance or budget	Users	99	89.2
	No sources	12	10.8
Mode of payment of water fee	Per-Container	5	4.5
	Daily	3	2.7
	Monthly	61	55.0
	Free	23	20.7
	Other	19	17.1
Amount of water fee per HH per month	Not set	36	32.4
	0.10–5.00	56	50.5
	>5–10.00	7	6.3
	>10	12	10.8
Water fee at committee members hand	None	76	68.5
	1.00–2000.00	32	28.8
	> 2000.00	3	2.7
Water fee not collected	Fee collected	107	96.4
	Not collected	4	3.6
Cash used for operation and maintenance	None	85	76.6
	Up to 2,000.00	9	8.1
	> 2000.00	17	15.3

## Discussion

This research suggests that if the goal of SDG 6.1 is to be achieved “universal access to safe drinking water for all” water quality parameters must be mandatory, not optional, as the current definitions state. This research suggests two potential pathways for monitoring global and country-level progress toward SDG 6.1 and access to clean drinking water. Both pathways emphasize the importance of water quality in understanding existing water services available to people around the world. Since the presence of TC and TTC is associated with negative health outcomes in the form of mortality and morbidity, it is essential that water quality is isolated as a variable to be addressed through the application of chlorine by maintaining the ‘pH’ and turbidity of the basic water that provides operational and verification of monitoring water quality parameters. Currently, water services classified as “basic” may lack nearby accessibility, potability, *or* availability. This means that multiple variables are collapsed through a classification of water services as “basic.” While additional detail has been captured by defining basic water services as functional or non-functional, this does little to provide actionable knowledge about the potability of water providing through basic water services. Water quality provides the strongest metric toward achieving SDG 6.1 goal of “safe drinking water” since achieving accessibility and/or availability water of poor quality does little to address the mortality and morbidity experienced by millions around the globe.

In the first suggested pathway, a water service must offer non-contaminated drinking water to be classified as “functional.” In other words, this suggested revision places a water quality parameter *inside* the definition of “functional” or “non-functional” water services. In current definitions, classification as functional or non-functional is based upon whether or not the water service operates to deliver water at the time of survey. See Table 11 for the suggested revisions to the SDG definitions.

**Table 11:** Suggested revisions to basic water services—Adding water quality parameters to functional and non-functional classifications.

	Functional Basic Water Service	Non-functional Basic Water Service
**SDG Definition**	If the water from improved source does not meet any **one** of the three criteria (source is accessible on premises, water is available on demand, water is free from contamination and a round trip to collect water takes 30 minutes or less including queuing.	If the water service from improved source fails to fully operate or only partially operates at time of survey.
	*Water quality optional*	*No water quality priority parameter considered*
**Suggested Revision**	If the water from improved source is free from contamination of priority parameters (TC and TTC/*E*. *coli*), **and** it is not on premise **or** available on demand, **and** a round trip to collect water takes 30 minutes or less including queuing.	If water service is fails to fully operate or only partially operates at time of survey **or** is not free from priority contaminants (TC and TTC/*E*. *coli*).
	*Priority water quality parameter mandatory*	*Priority water quality parameter mandatory*

A second potential pathway for improvement would be to alter the Drinking Water Ladders used in SDG Water Monitoring. In its current form, too many variables are collapsed into the definition of “basic water” services—policymakers and interested stakeholders do not know if the issue is water quality, availability, or accessibility. Adding a rung that specifies water quality with nearby (not on premises) accessibility will allow for better clarity and data capture around precisely how to target improvement programming, policies, and development schemes. See Table 12 for the suggested additional “safe basic water service” rung to be placed below “safely managed water service” but above “basic water service.”

**Table 12:** Suggested revisions to drinking water ladder rungs: Adding “Safe Basic Water Service”.

Ladder Rungs	Rung Definition
**Safely managed water service**	Drinking water from a source that is (1) accessible on premises **and** (2) free from contamination **and** (3) available on demand.
**Safe basic water service**	Drinking water from an improved source that is (1) accessible nearby so that a round trip to collect water takes 30 minutes or less including queuing **and** (2) free from contamination.
	If water is operationally available on demand, at time of survey, then it may be classified as functional.
	If water is not operationally available on demand, at time of survey, then it may be classified as non-functional.
**Basic water service**	If an improved source does not meet any **one** of the three criteria **and** a round trip to collect water is *less than* 30 minutes.
	If water is operationally available on demand, at time of survey, then it may be classified as functional.
	If water is not operationally available on demand, at time of survey, then it may be classified as non-functional.
**Limited water service**	If an improved source does not meet any **one** of the three criteria **and** a round trip to collect water *exceeds* 30 minutes.
**Unimproved water service**	Drinking water from an unprotected spring or dug well.
**Surface water**	Drinking water taken directly from a river, lake, pond, canal, irrigation channel or other.

This study does have limitations. As this study design was an observational cross-sectional, it was possible to determine if there were association between variables but not causal effects or inferences. Local government structures such as Zonal and Woreda Water Offices were not included in the study, which could help to substantiate findings through the triangulation of data from different sources. Future research could include these additions or expand this research design to new regions and woredas that experience less food insecurity.

## Conclusions

This research suggests that if the goal of SDG 6.1 is to be achieved “universal access to safe drinking water” water quality parameters must be mandatory, not optional, as the current definitions state. The findings suggest two potential pathways for improving data collection around water services, water quality, and determining the functionality of basic water services—including water quality as a parameter of “functionality” of a water service or by adding a rung titled “safe basic water service” on the Drinking Water Ladder for monitoring.

Our findings from Amhara and Afar regions of Ethiopia emphasize the importance of water quality to be added to the definition of basic water services in global development indicators. 38 (34.23%) of “functional” water services are reclassified as “non-functional” water services when we include water quality results—meaning this water is not “safe drinking water.” Speaking more broadly, for water and development specialists focused on achieving the Sustainable Development Goal 6 regarding global achievement of “clean water and sanitation,” our findings from Ethiopia suggest that additional research in other developmental contexts should be undertaken to assess the percentage of basic water services that, when water quality is taken into account, are found to be non-functional. Further, our findings around WASHCos demonstrate that research and developmental programming in other countries would do well to consider strengthening local management of water services and train local managerial committees in the importance of water quality and methods for water quality treatment.

## Supporting information

S1 File Survey questionnaire and FDG checklist. (PDF)

S2 File Laboratory protocol for bacteriological and physico-chemical technique in water sample analysis. (PDF)

S3 File Data of functionality and water quality assessment for basic water. (XLS)
