# Supplementary material for: The importance of water quality in classifying basic water services: The case of Ethiopia, SDG6.1, and safe drinking water
Source: PLoS One. 2021 Aug 5;16(8):e0248944. doi: 10.1371/journal.pone.0248944 (PMC8341575; doi:10.1371/journal.pone.0248944)
Supplement: S1 File — (PDF) [file pone.0248944.s001.pdf]

1. Laboratory protocol for bacteriological and physico-chemical technique in water sample analysis

1.1. Membrane filter technique (MF): International Organization for Standardisation (ISO) standard title (water quality): ISO: 9308-1:2014 Detection and enumeration of *Escherichia coli* and coliform bacteria. A technique that consists of filtering a water sample on a sterile filter with different pore sizes mostly recommended a 0.45-µm pore size using pressure that retains bacteria. Then incubating this filter on a selective medium and enumerating typical colonies on the filter by expressing in colony forming unit (CFU/100 ml).

The membrane filtration protocol applied for this research in the Ethiopian Public Health Institute (EPHI), Regional Lab is as the following.

1. We take water samples of 500 ml from the water sources in the study area in sterile water sample collection bottles.
2. The samples were labeled and transported to the EPHI laboratory, Dessie branch using cold box within 4 hours from the sample collection time for analysis.
3. The powder Membrane Lauryl Sulphate Broth (MLSB)-AVONCHEM-ACM-1820-O, lot number-B004113 of 38.1 gram per one liter for about 200 water samples was prepared using distilled water.
4. The bottles are loosely capped, placed in the autoclave and sterilized for 20 minutes at 121 °C using autoclave before use.
5. The water samples of 100 ml filtered through a sterile gamma membrane filter of Microdisc Filter of pore size 0.45 µm and size of 47 mm diameter after preparing the sample into the filter funnel up to the 100 ml graduation.
6. Samples were introduced aseptically into a sterile or properly disinfected filtration assembly.
7. The hand vacuum pump was used to filter and pump to suck the water sample through the membrane.
8. The membrane filters placed grid side up using sterilized forceps on GELMAN-filtration pad that had MLSB media.
9. Following a period of recovery (5 – 10 minutes), during which the bacteria become acclimatized to the new conditions, the Petri dish is transferred to an incubator.
10. The membrane filters on the media incubated at 37 ^{0}C for 24 hours for total coliforms (TC) and at 44 ^{0}C for 24 hours for Thermotolerant Coliforms (TTC).
11. Bacterial growth in water samples that brought from the improved water sources of each yellow color were counted per each Petridish and the results were registered in Colony Forming Units per 100 ml.
12. The bacterial colonies that grow on the filter papers per each medium were rolled safely, transported and disposed as recommended for the infectious waste disposal method.

1.2. Physcio-chemical test protocol: Palintest-Photometer-7100 instrument, which is product of Wagtech™ Potatech using photometer method is used to test the pH and free residual chlorine (FRC), and Palintest-turbimeter used for turbidity measurements. For testing pH, phenol red, free residual chlorine, diethyl-p-phenylene diamine (DPD) and for turbidity Turbimeter plus methods are used.

Test Instructions for Free Residual Chlorine (DPD)

1. Rinse test tube with sample leaving a few drops of sample in the tube.
2. Add, and then crush, the DPD No 1 tablet in the few drops of the water sample until the tablet is thoroughly crushed.
3. Add the 10 ml test solution, mix and seal the tube with the cap.
4. Fill test tube with double distilled water (blank) to the 10 ml mark
5. Select Phot 7 on Photometer.

6. First read the blank test and then the sample
7. Take Photometer reading in usual manner - see Photometer instructions.
8. The result represents the **free chlorine residual** in milligrams per litre (mg/l).

Test instructions for pH (Phenol red)

1. Fill test tube with sample to the 10 ml mark.
2. Add one Phenol Red tablet, crush and mix to dissolve.
3. Fill test tube with double distilled water (blank) to the 10 ml mark
4. Select Phot 27 on Photometer.
5. First read the blank test and then the sample
6. Take Photometer reading in usual manner (see Photometer instructions).

Notes

7. The colour range of the phenol red test is yellow, through orange, to red. The formation of an intense purple coloration shows that the indicator has been affected by high chlorine or other disinfectant residuals. In such cases the result should be disregarded.
8. Phenol red does not show any further colour change at pH values below 6.8 or above 8.4. Note therefore that when such values are recorded this could indicate that the sample has a much lower or much higher pH value.

Test instructions for turbidity

1. Fill test tube with sample to the 10 ml mark.
2. Fill test tube with double distilled water (blank) to the 10 ml mark
3. First read the blank test and then the sample using the turbimeter instrument
4. Take Turbimeter reading in usual manner (see Turbimeter instructions).
