# Supplementary material for: The importance of water quality in classifying basic water services: The case of Ethiopia, SDG6.1, and safe drinking water
Source: PLoS One. 2021 Aug 5;16(8):e0248944. doi: 10.1371/journal.pone.0248944 (PMC8341575; doi:10.1371/journal.pone.0248944)
Supplement: S2 File — (PDF) [file pone.0248944.s002.pdf]

Survey questionnaire for the study of determinants of basic water services functionality and contribution of contamination for non-functionality: towards SDG-6.1 in Ethiopia

Good morning/ good afternoon. My name is (please tell your full name). I am part of a team who are studying the determinants of basic water services functionality and contribution of contamination for non-functionality in your community. Your local leaders granted us permission to conduct this study. You were selected to answer some of our questions. If you agree to participate in the study, I will ask you questions about your drinking water conditions, the WASHCos management overall and will take samples of the water that you are drinking from the source for the purpose of laboratory examination. The interview will take approximately 15 minutes. The objective of the study is to examine the status of functionality of basic water services in your community by considering water quality as basic functionality factor.

1. WASHCo Location.	WASHCos contact person	Water scheme type	Status of water scheme
Woreda ____________<br>Kebele ____________<br>Village ____________<br>Establishment Date__________<br>No. of Beneficiary people (HH)Female(HH) ____ Male(HH) ____	Name ______________<br>Phone no.______________<br>Occupation ______________<br>Role In WASHCo______________<br>Sex:- Male ☐ Female ☐	On spot Spring ☐<br>Spring & RPS(Gravity) ☐<br>Spring/BH/(Motorized) ☐<br>HDW ☐<br>RWH ☐<br>New ☐ Rehab/ extension☐	Functional ☐<br>Partly functional ☐<br>Not functional ☐<br>No. of Active WASHcos,<br>Female ____ Male ____

2. If the water scheme is Not functional or encountered frequent failures?, What are the reasons.

Poor Management☐NoTechnician☐Unskilled Technician☐Lack of tools☐Lack of Spares☐ Lack of money☐

If any other reason, specify____

____

3. What is the objective of WASHCo establishment? ______________________________

____

____

4. How WASHCos were selected?____

____

5. Do WASHCo know their duties and accountability?Yes ☐ No☐

If No, Why? ____

6. Is there an active member during this assessment?

Yes ☐No ☐

Do WASHCo have regular meetings with water users?Yes☐ No ☐

If yes, in what frequency? ______________________

7. Was the WASHCO's gender composition maintained during the establishment?

Yes ☐ No ☐

Is it still maintained?

Yes ☐ No ☐

8. What is the role of females in the WASHCo?

Chairperson☐ V/Chair Person☐ Secretory☐ Casher☐ Caretaker☐Store keeper☐None☐

Other ____

**9.** Are the female WASHCO members actively discharging their role?<br><br>Yes ☐ No ☐	If not, what are the factors for women's weak participation?
**10.** Did theoretical and technical trainings been provided to the WASHCo members in the past?Yes ☐ No ☐<br>If yes; list the topics covered in training :- ______________________________	
**11.** Is there any sub committees assigned for each water points and reporting to the main WASHCos? Yes☐ No ☐<br>If yes, Explain their relationship/difference with main WASHCos? ______________________________	
**12.** Is there any experience in maintaining the operation of the facilities by their own? Yes ☐ No ☐<br>If yes, what type of maintenance work? Plumbing☐Masonry/concrete related☐Electrical ☐<br>Others? specify ______________________________	
**13.** How do the WASHCos access to spare parts? ______________________________	
**14.** Is there any controlling mechanism in and out spare parts? (In general materials management)? Yes ☐ No ☐<br>If yes, explain______________________________	
**15.** Is there any motivations given to the WASHCos? If yes, which type of motivations provided<br>Materials☐Trainings☐Review meeting☐Monitoring and supervision☐Others ____________<br>• From where/whommotivations provided ______________________________	
**16.** How do you value the Degree of cooperation or teamwork between members of the WASHCo?<br>Low ☐Fair ☐Good☐Very good☐ Excellent☐<br>• Explain If there any story of success or failureto learn from______________________________	
**17.** Do the WASHCos have a regular meeting (with minute) calendar?Yes☐ No☐<br>f yes, rate of meeting interval is ____________	
**18.** Is there a trend for community gathering & discussion with WASHCos? Yes☐ No☐<br>• If yes, explain when and in what circumstance? ______________________________	
**19.** Is there accountability of WASHCOs and reporting requirement (activities and financial) to the communities?<br>Yes☐ No☐explain______________________________	
**20.** How is the Documentation of WASHCOs? Which docs are available? (*Please take picture*)<br>Financial/management manuals☐ Administrative/bylaw manuals☐ Minutes☐ Beneficiaries list☐ Saving account☐<br>Others______________________________	
**21.** What is the role of WASHCos in disinfecting the water supply system? ______________________________	

22. When or at what time intervals the water be treated by chlorine and by whom?________________________________

____

23. How do the sanitation &hygiene promotion activity addressed in the kebele/ward in relation to latrine coverage (i.e., construction & proper utilization) and safe water handling practices? ____

____

____

24. Key achievements/success story in the O &M and further expansions of the WASH system since the establishment:_____________________

____

____

____

____

____

25. Major challenges faced since the WASHCo establishment: ____

____

____

26. Next plan to take forward the key achievements and overcome the challenges:____

____

____

27. **Financial Data**

- Sources of financing/budget are____
- How much Money available in Bank/microfinance account? _______________________________________
- Mode of payment of water users for water fee? Per Container☐ Daily☐weekly☐Monthly☐ Free☐
- Amount of water fee per household or Jerican (any other mode)? _________________________________
- Total amount of water fee collected so far __________ in Bank______ in committee members hand______ non collected from households_____________, others____

  ____
- How much of cash used for O &M so far? _________________
- How much of cash are deposited in bank? ________________

28. **Major Conclusions and recommendations (three per WASHCo):**

- ____
- ____
- ____

Name of study team participated in the assessment:

	**Name**	**Position**	**profession**	**signature**
1				
2				
3				

4				

Thank you!

Data Collector:-

Name_____________________ Position_____________________ Signature________________Date___________

FGD check lists for the study of determinants of basic water services functionality and contribution of contamination for non-functionality: towards SDG-6.1 in Ethiopia

1. Introduction of discussants and the objective of the study.
2. The status of the water service and the type of the water technology that the WASHCos are managing at this time.
3. What are the reasons for functionality or non-functionality of the water services they are managing?
4. How WASHCos are selected, established, their duties and responsibilities in a team or individually?
5. How do you evaluate the engagement of the WASHCos in their routine work, regular meetings and how is the gender composition?
6. What is and how do you see the role of females in the WASHCos? If they are not active what are the reasons?
7. Have you taken any capacity building trainings?
8. Could you tell us any experience of operation and maintenance carried out for the basic water services?
9. How do the WASHCos access to spare parts? Is there any controlling mechanism in and out spare parts?
10. How you are motivated for being a WASHCo members?
11. How do you value the degree of cooperation or teamwork between members of the WASHCo?
12. How do you address accountability of WASHCOs and reporting requirement (activities and financial) to the communities?
13. Key achievements/success story in the O &M and further expansions of the WASH system since the establishment
14. Major challenges faced since the WASHCo establishment and your next plan to take forward the key achievements and overcome the challenges

Thank you for your participation!
